# Comparison of perioperative outcomes in elderly (age ≧ 75 years) vs. younger men undergoing robot-assisted radical prostatectomy

**DOI:** 10.1371/journal.pone.0234113

**Published:** 2020-06-04

**Authors:** Yuta Yamada, Taro Teshima, Tetsuya Fujimura, Yusuke Sato, Masaki Nakamura, Aya Niimi, Naoki Kimura, Shigenori Kakutani, Taketo Kawai, Daisuke Yamada, Motofumi Suzuki, Haruki Kume

**Affiliations:** 1 Department of Urology, Graduate School of Medicine, The University of Tokyo, Bunkyo-ku, Tokyo, Japan; 2 Department of Urology, Jichi Medical University, Shimotsuke-shi, Tochigi-ken, Japan; 3 Department of Urology, National Center for Global Health, Shinjuku-ku, Tokyo, Japan; 4 Department of Urology, Mitsui Kinen Hospital, Tokyo, Japan; Brigham and Women's and Harvard Medical School, UNITED STATES

## Abstract

**Objectives:**

To investigate perioperative, oncologic, and functional outcomes of robot-assisted radical prostatectomy (RARP) in men of age ≥ 75 years in comparison with younger men.

**Methods:**

From November 2011 to December 2018, six hundred and thirty patients with prostate cancer underwent robot-assisted radical prostatectomy (RARP). A total of 614 patients were analyzed after excluding 16 patients who were treated with hormone therapy prior to RARP. Patients were divided into 2 groups based on their age (age ≥ 75 years: N = 46 patients and age < 75 years: N = 568 patients). Perioperative parameters regarding oncologic/functional outcomes and complication status were compared between the 2 groups. Clavien-Dindo classification was used to classify perioperative complications. Clinical and pathological status including stage, positive margin, continence, and potency status after RARP were analyzed.

**Results:**

Five-hundred sixty-eight and forty-six men were of age <75 and ≥ 75 years, respectively. There were no significant differences between the 2 groups in terms of oncologic outcomes (positive resection margin rate and PSA failure). The duration of hospitalization was longer in older patients but was not statistically significant (P = 0.051). A total number of Clavien ≥3 complications that occurred within a month after RARP were 15 (2.6%) and 2 (4.3%) in younger men (age < 75 years) and older men (age ≥ 75 years), respectively (P = 0.359).

**Conclusion:**

The present study showed that the oncologic and surgical outcomes in the elderly group were similar to those in the younger population. However, the duration of hospitalization seemed to be longer in older patients (age ≥ 75 years), despite similar complication rates.

## Introduction

Prostate cancer is the second most frequent malignancy among male worldwide and fourth common malignancy in Japanese men [1,2]. According to the latest annual statistical survey of Japan which was provided by the Ministry of Health, Labour and Welfare, life expectancy at birth were 80.98 years for males and 87.14 for females [2]. This number has been gradually incremented for the last 6 decades [2]. To urologists, this trend of reduction of mortality rates in the elderly population may inevitably generate an anguished decision in treatment option, since there is a common acceptance that the indication of radical prostatectomy (RP) is to be given to men under 70–75 years old [3]. Conversely, not all elderly men are unhealthy and intolerable to surgery. Besides, robot-assisted radical prostatectomy (RARP) has a better safety profile compared to open RP [4–6]. For this reason, it may be feasible to expand surgical indications of RARP to older men. In the RARP-era, such current circumstances raise the question “Is it acceptable not to recommend RARP to patients in elderly men?” The controversial aspect of this current circumstance is determining a patient selection of performing RARP in elderly men based on various elements.

One of the elements is life expectancy. In general, a life expectancy of 10 years is one standard in the decision-making of treatment types for prostate cancer [7,8]. There are several tools that could easily calculate the patients’ life expectancy [9,10]. However, most of these tools are not based on the presence or degree of comorbidities. Therefore, the surgeon should integrate different types of factors to make decisions on treatment options. For instance, besides the life expectancy, the surgeon must consider the clinical stage of prostate cancer, the patients’ most desired expectations (e.g. potency and continence), presence of comorbidities, and expected complications of the treatment. In elderly patients, treatment decisions may weigh more on the expected complications of the chosen type of treatment. This is because comorbidity rates are higher in the elderly population and feasibility may be more significant in these men. Therefore, investigating complication rates in these men undergoing RARP is important. Some reports investigated surgical or functional outcomes in men over 70 years [3,11–13]. However, fewer reports exist to explore safety and complication rates of RARP in much older men [14]. The purpose of this study was to investigate perioperative status regarding oncologic, surgical, and functional outcomes of RARP to provide supportive information on decision making for performing RARP in elderly men with prostate cancer.

## Materials and methods

### Patient characteristics

Between November 2011 and December 2017, six hundred and thirty consecutive patients underwent RARP for prostate cancer at a single institution. Sixteen patients were excluded from the study, given that they were treated with hormone therapy prior to RARP, which allowed us to investigate 614 patients in total. We retrospectively investigated complications and comorbidity of these patients and added to our prospectively collected database currently used in our previous literature [15]. PSA failure was defined as 2 consecutive elevation of PSA values above 0.2 ng/mL. Patients were asked to remember the date when he accomplished 1 pad or less per day or pad free. This date was prospectively collected in our database at the next visit. Cancer staging was based on the American Joint Committee on Cancer (AJCC) TNM staging system [16]. Tolerability and risk assessment of surgery were determined by the attending surgeons according to preoperative tests including blood tests, electrocardiogram, chest/abdomen x rays, spirogram, and echocardiography when necessary. Preoperatively, patients with ASA score ≥ 3 were referred to anesthesiologists or specialists that were involved with the specific comorbidity to evaluate the tolerability of undergoing surgery. Patients with glaucoma with closed primary angle, severe heart failure, or previous history of surgery involving rectal cancer were not recommended for RARP procedure. Routine follow-ups were done at our outpatient office after discharge at 2 weeks, 1, 3, 6, 12 months, and 6–12 months cycle thereafter, unless complications occurred that require visits. Patients were followed for at least 6 months in this study. Complications were categorized based on the Clavien-Dindo classification [17,18].

All patients provided written informed consent to have their medical records used in the research and our study was approved by the institutional review board “Ethics Committee of the Tokyo University Hospital” (#3124). This study is following the Helsinki declaration. All data were anonymized after completion of collecting the data. The data range was between November 2011 to October 2018. The data used for the present study was directly collected from the medical records used in The University of Tokyo Hospital.

### Surgical technique

All patients underwent RARP using the da Vinci surgical robot system (da Vinci-S or Xi^®^: Intuitive Surgical Incorporation, Sunnyvale, CA). All surgeries were carried out using the transperitoneal, six-port technique, as described in our previous studies [19,20]. Briefly, bladder neck dissection was performed following the dissection of the seminal vesicles. Dorsal vein complex (DVC) was cut by scissors and was vertically sutured by a 3–0 absorbable monofilament. Nerve-sparing was performed in indicated patients. The urethra was cut at the level of the apex of the prostate. Reinforcement of the pelvic floor was carried out by using Rocco’s stitch [21] following the resection of the prostate. Urethro-vesico anastomosis was carried out using a 3–0 absorbable monofilament. By using this surgical method, a total of 19 surgeons performed RARP in the present study.

### Statistical analyses

We used statistical software JMP^®^ Pro version 14 (© SAS Institute Inc., Cary, CA) for statistical analysis. Student’s t-tests were used to compare continuous values between the 2 groups (age ≥ 75 and < 75 years). The Pearson’s chi-square test (χ^2^) and Fisher test were used for analysis of categorical variables. We performed the Cochran–Armitage trend test to analyze the inherent trend among Clavien grades and age groups. Kaplan-Meier curves with a log-rank test was performed to compare the time to achieving continence or prostate-specific antigen (PSA) free survival in the 2 groups. The secondary cutoff of age was also determined according to the median value (68 years-old) to compare the outcomes between age groups regarding the recovery of continence. P value of <0.05 was considered statistically significant.

## Results

Demographics of 614 patients with prostate cancer who underwent RARP are shown in Table 1. A total of 568 men were < 75 years and 46 men were aged ≥ 75 years. The median follow-up was 33.6 (interquartile range: 20.4–48.9) months, average follow-up was 35.2 months. Higher ASA scores were observed in older men (≥ 75 years) (P = 0.002, Table 1). There were no statistically significant differences in other clinical backgrounds including clinical T stage and D’Amico risk classification (Table 1).

**Table 1:** Baseline patient characteristics (N = 614).

Variable		Mean value ± SD or number of cases (%)		
		Age < 75 years (N = 568)	Age ≥ 75 years (N = 46)	P value
Age (year)		66.0 ± 5.7	75.7 ± 1.2	<0.001
BMI (kg / m^2^) (N = 604)		24.0 ± 2.9	23.7 ± 2.7	0.521
Preop- PSA (ng / ml)		9.8 ± 7.9	10.2 ± 5.6	0.693
Preop- PV (cm^3^) (N = 613)		32.0 ± 15.6	31.2 ± 16.4	0.713
Preop- Gleason score	6	102 (18.0)	6 (13.0)	0.475
	7	322 (56.7)	25 (54.4)	
	≥8	144 (25.3)	15 (32.6)	
ASA scores	<3	530 (93.3)	37 (80.4)	0.002
	≥ 3	38 (6.7)	9 (19.6)	
Clinical T stage	cT1c	446 (78.5)	33 (71.7)	0.285
	cT2a-cT3	122 (21.5)	13 (28.3)	
D’Amico risk classification	Low	75 (13.2)	6 (13.1)	0.422
	Intermediate	322 (56.7)	22 (47.8)	
	High	171 (30.1)	18 (39.1)	

BMI: body mass index, Preop: preoperative, PSA: prostate specific antigen, PV: prostate volume, ASA: American Society of Anesthesia. Student’s t tests were used in continuous values and Pearson’s chi square tests were used in categorical values. P value < 0.05 was considered as statistically significant.

Preservation of the neurovascular bundle was performed more frequently in younger (< 75 years) men (P = 0.003, Table 2). There were no significant differences in surgical and oncological outcomes. Specifically, positive margin rates in pT2 patients were 13.6% and 7.1% in younger (< 75 years) and older men (≥ 75 years), respectively (P = 0.560, Table 2).

**Table 2:** Surgical and oncological outcomes of RARP (N = 614).

Variables		Mean value ± SD or number of cases (%)		
		Age < 75 years (N = 568)	Age ≥ 75 years (N = 46)	P value
NVB preservation	None	397 (69.9)	43 (93.5)	0.003
	Unilateral	163 (28.7)	3 (6.5)	
	Bilateral	8 (1.4)	0 (0)	
Operative time (min.)		231 ± 61	231 ± 60	0.967
Console time (min.)		174 ± 54	170 ± 53	0.560
Estimated blood loss (ml.)		371 ± 369	358 ± 342	0.811
pT stage	≤ pT2a-T2c	381 (67.1)	28 (60.9)	0.391
	≥ pT3a	187 (32.9)	18 (39.1)	
pN stage (N = 139)	N0	115 (91.3)	12 (92.3)	0.899
	≥ N1	11 (8.7)	1 (7.7)	
Extraprostatic extension	Absent	391 (68.8)	28 (60.9)	0.264
	Present	177 (31.2)	18 (39.1)	
Resection margin	Negative	329 (86.4)	26 (92.9)	0.560
(≤ pT2 patients)	Positive	52 (13.6)	2 (7.1)	
Resection margin	Negative	104 (55.6)	11 (61.1)	0.654
(≥ pT3 patients)	Positive	83 (44.4)	7 (38.9)	
SV invasion (N = 613)	Negative	513 (90.5)	42 (91.3)	1.000
	Positive	54 (9.5)	4 (8.7)	

SD: standard deviation, NVB: neurovascular bundle, SV: seminal vesicle. Student’s t tests were used in continuous values. Pearson’s chi square tests or Fisher’s tests were used in categorical values. P value < 0.05 was considered statistically significant.

**Fig 1:**
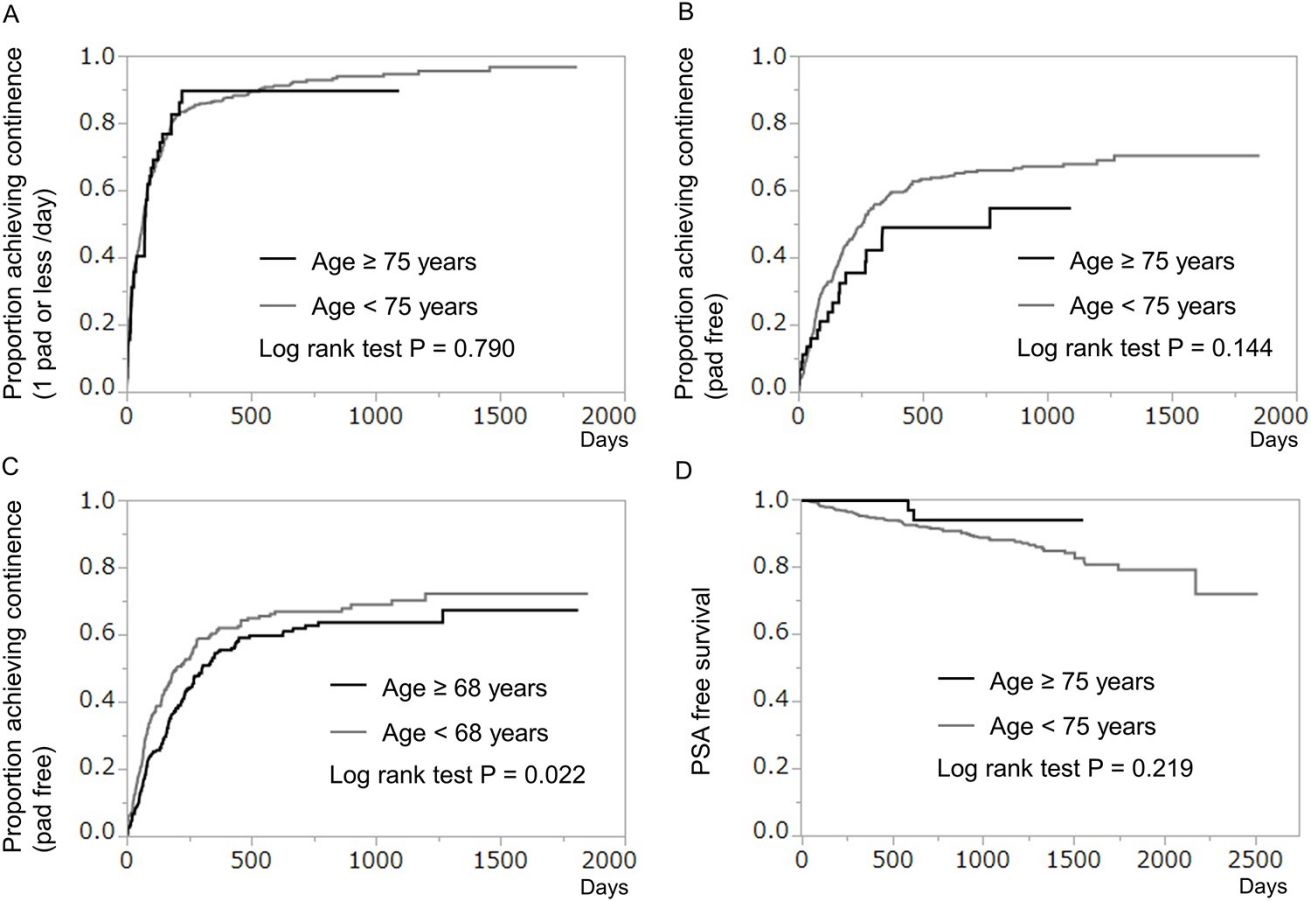
A-C: Kaplan-Meier curves showing cumulative proportion of achieving continence after robot-assisted radical prostatectomy (RARP). D: Kaplan Meier curve showing PSA free survival in patients who underwent RARP. (A) Proportion of patients achieving 1 pad or less/day for urinary continence. There were no significant differences between patients of age ≥ 75 years and patients of age < 75 years (log-rank test P = 0.790). (B) Proportion of patients achieving pad-free status for urinary continence. There were no significant differences between patients of age ≥ 75 years and patients of age < 75 years (log-rank test P = 0.144). (C) Proportion of patients achieving pad-free status for urinary continence in men of age ≥ 68 years and < 68 years. Younger patients (age < 68 years) had a significantly higher recovery rate (log-rank test P = 0.022). (D) There were no statistically significant differences regarding PSA free survival between the two groups (log-rank test P = 0.219).

Kaplan -Meier estimates of continence recovery (1 pad or less /day) after RARP at 12 months were 86.5% and 89.5% in younger (< 75 years) and older men (≥ 75 years), respectively (P = 0.790, Fig 1A). In terms of pad free rates, Kaplan -Meier estimates of continence recovery were 58.4% and 48.9% in men of age ≥75 years and <75 years, respectively (P = 0.144, Fig 1B). However, when cutoff-line was set to 68 years old, continence recovery rates (pad free) were higher in younger (< 68 years) men (P = 0.022, Fig 1C). There was no significant difference regarding PSA free survival between men of age ≥75 years and <75 years (P = 0.219, Fig 1D).

The duration of hospitalization was longer in older men but was not statistically significant (P = 0.051, Table 3). There were no significant differences between the two groups in terms of urinary leakage, duration of catheterization, and perioperative complications (Table 3). Specifically, total numbers of Clavien ≥3 complications were 15 (2.6%) and 2 (4.3%) in younger and older men, respectively (Table 3).

**Table 3:** Perioperative outcomes including complications occurring within 1 month (N = 614).

Variables	Mean ± SD or number of cases (%)		
	Age < 75 years	Age ≥ 75 years	P value
Urinary leakage (postop-cystogram)	29 (5.1)	2 (4.4)	1.000
Blood transfusion	23(4.1)	4 (8.7)	0.136
Duration of catheterization (days)	7.2 ± 2.6	7.5 ± 3.5	0.421
Duration of hospitalization (days)	11.0 ± 3.6	12.1 ± 3.6	0.051
Perioperative complications *^1^			
No complications	426 (75.0)	32 (69.6)	0.352
Clavien 1	69 (12.2)	6 (13.0)	-
Clavien 2	58 (10.2)	6 (13.0)	-
Clavien 3a	3 (0.5)	1 (2.2)	-
Clavien 3b	11 (1.9)	1 (2.2)	-
Calvien 5 ^†^	1 (0.2)	0	-
Total number of all complications	142 (25.0)	14 (30.4)	0.415
Total number of Clavien ≥3 complications	15 (2.6)	2 (4.3)	0.369
Total number of complications during hospitalization	127 (22.4)	13 (28.3)	0.359

All complications were classified according to the Clavien-Dindo classification. SD: standard deviation, postop: postoperative.

^†^ One patient developed non-occlusive mesenteric ischemia and died on postoperative day 35 due to severe infection and multiple organ dysfunction. Chi-square test was used to compare statistical differences between categorical variables, and Student’s t tests were used for continuous variables.

*^1^ Cochran-Armitage trend test was performed for analysis regarding Clavien grades between age groups.

In a more detailed evaluation of the Clavien ≥3 complications that occurred within a month after RARP, postoperative hemorrhage was the only complication that occurred in older men (Table 4). One patient with non-occlusive mesenteric ischemia developed severe infection and multiple organ disease and died after 35 days (Table 4).

**Table 4:** Detailed Clavien ≥3 complications of RARP occurring within 1 month (N = 614).

Variables	Number of cases	
	Age < 75 years	Age ≥ 75 years
Bladder injury	1	0
Drain tube removal	1	0
Incisional hernia	2	0
Non-occlusive mesenteric ischemia ^†^	1	0
Postoperative hemorrhage	3	2
Rectal / bowel injury	4	0
Ureteral injury	1	0
Urinary leakage	2	0
Total	15	2

RARP: robot assisted radical prostatectomy.

^†^ One patient developed multiple organ disfunction due to non-occlusive mesenteric ischemia and died after 35 days.

## Discussion

The present study shows the comparison of perioperative parameters between elderly and younger men undergoing RARP procedure. ASA scores were higher in the elderly group. Nevertheless, no significant differences in the complication rates were observed between the 2 groups. Similar outcomes were also noted for oncologic parameters. Younger men seemed to be superior in terms of recovery of continence.

The determination of treatment modalities in elderly men with prostate cancer is complicated. Specifically, cancer control, functional outcome, tolerability to surgery, cost benefits, and patient preference are some of the fundamental elements that should be put into parts of the equation when selecting patients for robot-assisted radical prostatectomy (RARP) as a treatment in prostate cancer [22,23]. Besides, there are only 2 retrospective studies that evaluated surgical implications in men aged ≥ 75 years [14,24]. However, both studies were not specifically focused on the comparison between men aged ≥ 75 years versus younger men. One study by Pierorazio PM et al. was a report of data consisting of the entire cohort and not a compared study by age [24]. The other study compared surgical and functional outcomes in patients aged ≥ 75 years versus those in the overall cohort [14]. In this study, there were no significant differences between elderly and younger men regarding positive-margin and complication rates.

Life expectancy is an important factor on decision-making regarding treatment choice for prostate cancer. Based on the Japanese national survey shown by the Ministry of Health, Labour and Welfare, the life expectancy of a 75 and 80year-old male is 12.18 and 8.95 years, respectively [2]. Together with the NCCN [7] and JUA [8] guidelines recommending 10-year life expectancy as the key element of the inclusion criteria in performing RARP, it seems quite acceptable that patients whose ages are between 75–80 years-old are potential candidates of RARP procedure. Nevertheless, some urologists do not offer radical prostatectomy to patients over 70 years old due to the perception of elevated complication rates. The present study shows that RARP seems to be a safe treatment option with a surprisingly few major complications in a subset of elderly men.

RARP may offer many advantages over other treatment modalities. First, it seems that RARP provides superior cancer control to other treatment modalities [25,26]. A randomized trial comparing RP with watchful waiting in prostate cancer showed that RP reduced cancer-specific mortality [25]. A meta-analysis indicated that increased overall and cancer-specific mortalities were observed in patients treated with radiotherapy compared with those who underwent surgery [26]. Second, surgical specimens obtained from the RARP procedure provide accurate information on oncologic status, which may assist in the determination of adjuvant or salvage treatment choice. Finally, hormone therapy may substantially be more expensive than RP when it is carried out in the long term [27].

Overall complication rates in RARP procedure vary among studies [28–30]. In the present study, the complication rates showed similar results compared with the previous report by Alvin LW et al. who showed that the overall complication rate was 21.5% in RARP procedure [28]. Another study reported an overall complication rate of 12% in the initial 200 cases [29]. Specifically, major complication rates seem to be around 2–3% in most of the studies [28–30].

As expected, admission duration tended to be longer in older men. It seems that complications were not the reason, and there seems to be no valid explanation for the longer admission period for older men. However, according to our data, RARP performed on men aged ≥ 75 years compared with younger men did not translate into significantly worse oncologic and functional outcomes. In addition, complications over Clavien grade 3 were mostly surgical-related and had no direct relationship with preoperative comorbidities. Interestingly, according to the data given by Lee JY et al., Charlson Comorbidity Index had no impact on cancer-specific survival in 336 men who underwent RP for prostate cancer, although it was independently associated with overall survival [31]. A safety profile of RARP was reported by Agarwal PK et al., who pointed out that most of the complications in RARP patients were surgical complications (289 out of 368 complications) [32]. These findings suggest that safety in performing RARP may not be significantly influenced by preoperative comorbidities, but rather have an association with surgical factors.

Patient preference is one of the fundamental elements in determining treatment modality. In a study evaluating patient preferences for management of early localized prostate cancer, patients selecting surgical intervention were strongly influenced by the possibility of complete tumor removal with surgery, despite after explained about the set of complications including 1 to 2% chance of death [33]. If safety or tolerability is within the acceptable range, performing RARP in these patients may provide further mental stability and assurance. It may also influence more patients to prefer surgery.

There are several limitations to address. One, careful interpretation of this study is required, since it may have the selection bias in the surgeon’s mind. Surgeons may have recommended RARP to patients based on parameters other than ASA or comorbidities. Although we did not use geriatric screening tools such as G8 or VES-13 [34], the surgeons may have recommended RARP partly from the general condition of the patient himself. It may be effective to use such tools in the decision-making of treatment options. Second, there is a statistical small power of the population in the elderly age group and the lack of multivariable modeling. Third, the present study also lacks to compare the outcomes between other different treatment modalities in elderly men with prostate cancer. Fourth, the potential bias may include the surgeons’ relevant experience. Finally, the present study cannot provide analysis regarding the quality of life (QOL) and its association with RARP. QOL is important to elderly men as well as surgical or complication status and therefore needs to be evaluated in the future studies.

In conclusion, a simple strategic determination of treatment options by age alone seems to be arbitrary. It seems that RARP may be feasible in the elderly men although preoperative ASA classification scores were significantly higher. However, age seems to be a risk factor for delay in the recovery of continence. Therefore, it is important to address this issue and provide explanation regarding this matter to patients preoperatively. To determine treatment modalities in the elderly patients, further studies are necessary to evaluate the differences among treatment modalities by multifaceted perspectives regarding cancer control, complication rates, and cost benefits.

## Supporting information

S1 Data (XLSX)
